# Association between postdischarge antibiotic use and *C. difficile* testing as a surrogate for clinically significant diarrhea

**DOI:** 10.1017/ash.2025.10162

**Published:** 2025-10-16

**Authors:** Daniel J. Livorsi, James Merchant, Hyunkeun Cho, Matthew B. Goetz, Bruce Alexander, Michihiko Goto

**Affiliations:** 1 Center for Access and Delivery Research and Evaluation, Iowa City Veterans Affairs Health Care System, Iowa City, IA, USA; 2 Division of Infectious Diseases, University of Iowa Carver College of Medicine, Iowa City, IA, USA; 3 Department of Biostatistics, University of Iowa, Iowa City, IA, USA; 4 VA Greater Los Angeles Healthcare System, Los Angeles, CA, USA; 5 David Geffen School of Medicine at the University of California, Los Angeles, CA, USA

## Abstract

**Objective:**

Postdischarge antibiotics are often sub-optimal or unnecessary. This study sought to measure the risk of diarrhea in recently hospitalized patients treated with postdischarge antibiotics

**Design:**

Retrospective cohort study.

**Setting:**

125 acute-care hospitals in the Veterans Health Administration (VHA).

**Patients:**

Patients hospitalized within VHA during 2018–2021.

**Methods:**

The primary exposure was postdischarge antibiotics. The primary outcome was time to *C. difficile* testing, which served as a surrogate marker for clinically significant diarrhea. Only tests that were performed during the 30 days after discharge and before all-cause hospital readmission were captured. We constructed a final Cox proportional hazards model with 27 fixed-effect predictors as well as a random intercept for each hospital.

**Results:**

There were 1,686,819 qualifying admissions, and 333,310 (19.8%) received postdischarge antibiotics. There were 13,387 patients (0.8%) who had a test for *C. difficile* done. Among those tested, the median time to testing was 6.7 days for those tested while on postdischarge antibiotics and 14.1 days for those tested while not on postdischarge antibiotics. Compared to patients not on postdischarge antibiotics, the hazard ratio for testing was 1.40 (95% CI, 1.29–1.51) among patients on low-risk postdischarge antibiotics and 1.56 (95% CI, 1.42–1.71) among those on high-risk postdischarge antibiotics.

**Conclusions:**

In this national VHA hospital cohort, patients prescribed postdischarge antibiotics had a 40–56% increased risk of *C. difficile* testing compared to those not prescribed postdischarge antibiotics. Efforts to optimize antibiotic-prescribing at hospital discharge, particularly by reducing excessive duration and avoiding high-risk agents, may help mitigate these risks.

## Introduction

One out of every five patients discharged from an acute-care hospital is prescribed antibiotics to take after their hospital stay.^1^ Prior studies have found that 40–80% of these antibiotic prescriptions are either unnecessary or sub-optimal.^2–6^ Several hospitals have reported on their local initiatives to optimize antibiotic-prescribing at discharge.^3,7–11^

Improving antibiotic-prescribing at discharge may improve patients’ clinical outcomes, e.g., by reducing antibiotic-related adverse events. However, these antibiotic-related harms have not been well quantified. Determining the frequency at which postdischarge antibiotics are associated with adverse events may motivate further efforts to improve postdischarge antibiotic use.

A common antibiotic-related adverse event is diarrhea.^12^ The development of diarrhea, especially in patients recently hospitalized and/or treated with antibiotics, raises concern for *Clostridioides difficile* infection. In patients with a compatible clinical syndrome, diagnosing a *C. difficile* infection requires laboratory detection of either *C. difficile* toxins or toxigenic *C. difficile* organisms.

In this study, we used testing for *C. difficile* as a surrogate marker for clinically significant diarrhea. We sought to measure the risk of diarrhea in recently hospitalized patients treated with postdischarge antibiotics vs those who were not treated, after adjusting for other key confounders.

## Methods

This was a retrospective cohort study of all patients hospitalized to an acute-care hospital across 125 VHA hospitals between January 1, 2018 and December 31, 2021. We excluded patients who were transferred to another healthcare facility or to post-acute care in VHA, left the hospital before medically advised, were tested for *C. difficile* prior to discharge, or died while hospitalized*.*

### Data sources

We accessed national administration data from the VHA Corporate Data Warehouse (CDW) via the VHA Informatics and Computing Infrastructure (VINCI). Medication data was identified in the Barcode Medication Administration (BCMA) pharmacy data domain of the CDW and from outpatient medication files.

For each patient-admission, we collected data on all inpatient and postdischarge antibacterials (hereafter “antibiotics”) included in the National Healthcare Safety Network (NHSN)’s Antimicrobial Use and Resistance Protocol.^13^ This included inpatient antibiotics administered via the following routes: intravenous, intramuscular, digestive tract (eg, oral), or respiratory tract. Postdischarge antibiotics were defined as oral antibiotics dispensed from the outpatient pharmacy during the discharge period.^1^ We assumed that all outpatient oral antibiotics dispensed during this time frame were initiated by the patient on the day following discharge and were taken for a duration equal to the days-supply of the dispensed prescription. If more than one antibiotic was prescribed at discharge, we assumed all agents were taken concurrently.

### Variables

The primary exposure of interest was postdischarge antibiotics. Each day after discharge that the patient took the postdischarge antibiotic, assuming the antibiotic was taken as prescribed, was considered a day of postdischarge antibiotic exposure. Postdischarge antibiotics were classified into two categories: (1) agents posing the highest risk for *C. difficile* infection, and (2) all other agents.^13^ According to NHSN, agents with the highest risk for *C. difficile* infection are cefdinir, cefepime, cefixime, cefotaxime, cefpodoxime, ceftazidime, ceftriaxone, ciprofloxacin, clindamycin, gemifloxacin, levofloxacin, and moxifloxacin.^13^

The primary outcome was time to *C. difficile* testing. This test served as a surrogate marker for clinically significant diarrhea. In the primary analysis, only tests that were performed during the 30 days after discharge and before all-cause hospital readmission were captured. We chose not to use ICD-10 codes to measure diarrhea occurrence because these codes were used in <0.1% of all patients during the 30 days after hospital discharge.

We created a list of risk-adjustment variables at the time of the hospital discharge, including patient age, sex, hospital service at discharge, indicator variables for comorbid conditions, and body mass index (BMI, which was calculated as weight in kilograms divided by height in meters squared). Data on comorbid conditions were collected using a modified version of the Elixhauser comorbidity index, which identified patients as having comorbidities based on International Classification of Diseases, 10^th^ revision (ICD-10) codes from outpatient and inpatient encounters over the 12 months prior to the index visit and from the index admission itself.^14^ We also collected data on exposure to proton pump inhibitors (dexlansoprazole, esomeprazole, lansoprazole, omeprazole, pantoprazole, or rabeprazole), and histamine H2-receptor antagonists (famotidine, cimetidine, nizatidine, or ranitidine) during the hospital stay and at the time of hospital discharge. Finally, we collected data on length of hospital stay and inpatient antibiotic exposure, which we classified into two categories: 1) agents posing the highest risk for *C. difficile* infection, and 2) all other agents.^13^

### Statistical analysis

To address the time-dependent nature of postdischarge antibiotic use, we created two time-dependent variables. First, we developed an indicator variable for postdischarge antibiotic use that equaled 1 when the patient was scheduled to receive postdischarge antibiotics on that day, based on their prescription information; if no postdischarge antibiotics were scheduled for that day, this indicator variable was set to zero. A second time-dependent indicator variable was used to capture any possible residual effects of postdischarge antibiotic exposure during the 7-day period after postdischarge antibiotic use ceased. If patients were readmitted for any reason within 30 days, patient information for that visit was censored at the day of readmission. If patients died within 30 days, patient information was censored at the time of death.

We fit a Cox proportional hazards model to evaluate the association between postdischarge antibiotic use and time to *C. difficile* testing. Model selection was performed using backward elimination based on the Akaike Information Criterion (AIC). Certain variables deemed essential to the analysis were forced into the model: an indicator variable for total days of exposure to inpatient antibiotics and separately postdischarge antibiotics, a high-risk antibiotic interaction term (as defined by receipt of the above-mentioned agents), and length of hospital stay. In addition, 22 relevant comorbidity and demographic variables were considered as candidate variables: age, gender, BMI, gastric acid suppressive agents, medical vs surgical service prior to discharge, alcohol use disorder, anemia, arrhythmia, chronic pancreatitis, chronic pulmonary disease, congestive heart failure, diabetes mellitus, immunodeficiency condition, immunosuppressive medications, inflammatory bowel disease, liver disease, metastatic cancer, neurological disorders, peripheral vascular disease, pulmonary circulation disorders, renal failure and/or dialysis, and weight loss. Based on AIC, all candidate variables were retained in the final model. The resulting Cox model included 27 fixed-effect predictors and a random intercept for each medical center to account for between-hospital variability in antibiotic-prescribing and *C. difficile* testing practices.

Estimates of hazard ratios and confidence intervals for the four antibiotic groups of interest (low-risk antibiotic use, high-risk use, postantibiotic effect among low-risk antibiotic users, and postantibiotic use among high-risk antibiotic users) were produced via contrast statements for the appropriate combination of the antibiotic indicator, postantibiotic use indicator, and high-risk indicator variables. Using the variables selected for the primary outcome, similar time-dependent Cox proportional hazards models were also run on subsets of the data: 1) patients who were 65 years of age or older at discharge (*n* = 1,117,262) and 2) patients receiving gastric acid suppressive agents (*n* = 799,581).

## Results

**Figure 1.**
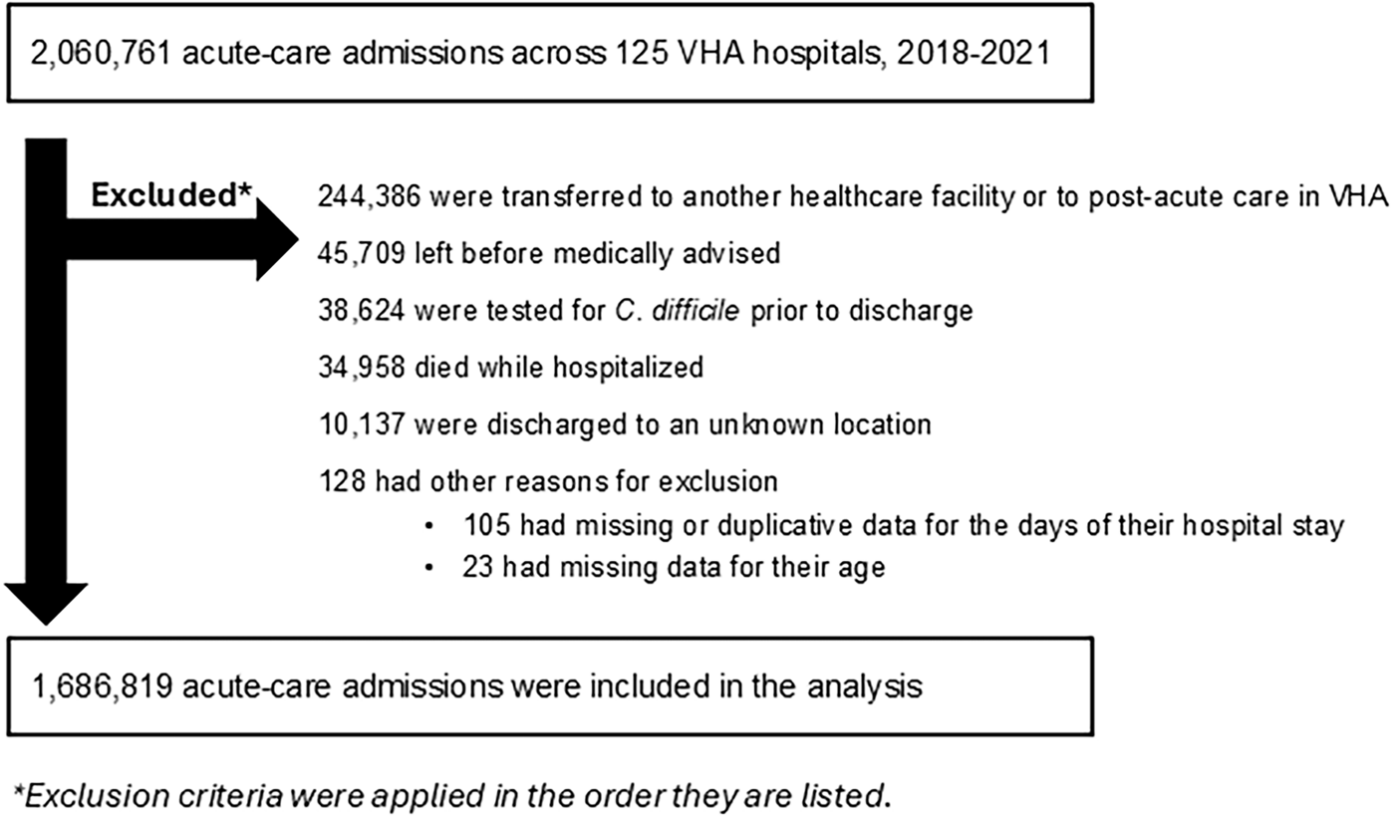
Flowchart for how the study cohort was constructed.

There were 2,060,761 acute-care admissions during 2018–2021, but 244,386 were transferred to another healthcare facility, 45,709 left before medically advised, 38,624 were tested for *C. difficile* prior to discharge, 34,958 died in the hospital, 10,137 were discharged to an unknown location, and 128 were excluded for other reasons (Figure 1). This left 1,686,819 admissions to include in our analysis. Table 1 summarizes the characteristics of these patient admissions. There were 333,310 (19.8%) who received postdischarge antibiotics and 1,353,509 (80.2%) who did not; 122,347 (36.7%) of the postdischarge antibiotics were classified as agents posing the highest risk for *C. difficile* infection. The median duration of postdischarge antibiotics was 6 days (4–10).

**Table 1.** Baseline characteristics of hospitalized patients exposed and not exposed to antibiotics at discharge, 2018–2021

	Total (*n* = 1,686,819)	Postdischarge antibiotics (*n* = 333,310)	No postdischarge antibiotics (*n* = 1,353,509)
Age, median (IQR)	70 (61-75)	70 (62-75)	69 (61-75)
Male sex, *n* (%)	1,581,358 (93.7)	313,779 (94.1)	1,267,579 (93.7)
Medicine specialty at discharge, *n* (%)	1,344,955 (79.7)	270,275 (81.1)	1,074,680 (79.4)
Length of stay, median days (IQR)	2 (1-5)	3 (2-4)	2 (1-5)
Inpatient antibiotic exposure, *n* (%)	685,831 (40.7)	310,986 (93.3)	374,845 (27.7)
High-risk inpatient antibiotic exposure	321,768 (19.1)	172,698 (51.8)	149,070 (11.0)
Gastric acid suppression, *n* (%)	799,866 (47.4)	153,048 (45.9)	646,818 (47.8)
Body mass index, *n* (%)^1^			
Underweight	35,555 (2.1)	8,057 (2.4)	27,498 (2.0)
Normal	389,210 (23.1)	78,356 (23.5)	310,854 (23.0)
Overweight	493,045 (29.2)	95,532 (28.7)	397,513 (29.4)
Obese	639,933 (37.9)	127,174 (38.2)	512,759 (37.9)
Missing	129,076 (7.7)	24,191 (7.3)	104,885 (7.7)
Chemotherapy, *n* (%)^2^	2,538 (0.2)	604 (0.2)	1,934 (0.1)
**Comorbidities, *n* (%)**			
Alcohol use disorder	317,902 (18.9)	52,524 (15.8)	265,378 (19.6)
Anemia	329,298 (19.5)	62,085 (18.6)	267,213 (19.7)
Cardiac arrhythmia	741,373 (44.0)	137,567 (41.3)	603,806 (44.6)
CHF	525,392 (31.1)	93,923 (28.2)	431,469(31.9)
Chronic pancreatitis	29,474 (1.7)	4,103 (1.2)	25,371 (1.9)
Chronic pulmonary disease	640,579 (38.0)	147,084 (44.1)	493,495 (36.5)
Diabetes mellitus	761,825 (45.2)	153,277 (46.0)	608,548 (45.0)
Inflammatory bowel disease	33,500 (2.0)	7,215 (2.2)	26,285 (1.9)
Immunosuppressive condition^3^	86,217 (5.1)	20,701 (6.2)	65,516 (4.8)
Liver disease	290,694 (17.2)	54,066 (16.2)	236,628 (17.5)
Metastatic cancer	100,020 (5.9)	19,950 (6.0)	80,070 (5.9)
Neurological disorders^4^	344,208 (20.4)	62,622 (18.8)	281,586 (20.8)
Pulmonary circulation disorders	86,325 (5.1)	16,042 (4.8)	70,283 (5.2)
Peripheral vascular disease	385,259 (22.8)	77,486 (23.3)	307,773 (22.7)
Renal disease and/or dialysis	472,378 (28.0)	89,073 (26.7)	383,305 (28.3)
Weight loss	224,686 (13.3)	44,796 (13.4)	179,890 (13.3)

1. Body mass index was classified as underweight (<18.5), normal (18.5–24.9), overweight or obese (25.0 and higher), and missing.

2. Chemotherapy was included if it was administered within 30 days before the admission or during the admission itself.

3. Immunocompromising diagnoses included lymphoma, leukemia, HIV, and organ transplantation.

4. This category included dementia, paralysis, paresis, Parkinson’s disease, multiple sclerosis, epilepsy, and other neurological disorders.

There were 252,293 patients (15.0%) who were censored, with censoring defined as a) hospital readmission within 30 days without subsequent *C. difficile* testing (*n* = 205,647); b) hospital readmission within 30 days with subsequent *C. difficile* testing (*n* = 5,814); or c) death before testing and before the 30-day study period ended (*n* = 40,832)

There were 13,387 patients (0.8%) who had a test for *C. difficile* done prior to censoring, including 1,260 tested while on postdischarge antibiotics and 12,127 tested while not on postdischarge antibiotics; 504 (40.0%) of the patients tested while on postdischarge antibiotics were on high-risk agents. The *C. difficile* tests that were performed included 10,445 (78.0%) PCR-based tests, 1,682 (12.6%) EIA-toxin tests, 978 (7.3%) two-step tests, and 282 (2.1%) tests of unknown methodology. Among those tested, the median time to testing was 6.7 days for those tested while on postdischarge antibiotics and 14.1 days for those tested while not on postdischarge antibiotics; 2,952 (22.1%) tests were positive for *C. difficile*.

Table 2 summarizes the multivariable Cox regression model. The hazard ratio for patients on low-risk postdischarge antibiotics relative to patients not on postdischarge antibiotics was 1.40 (95% CI, 1.29–1.51; *P* < .01). This indicates that for patients on low-risk postdischarge antibiotics, the risk of *C. difficile* testing during the observation period was estimated to be 40% higher than patients not on postdischarge antibiotics, holding other covariates constant. The hazard ratio for these low-risk antibiotic patients in the 7 days after the conclusion of antibiotics (relative to non-antibiotic patients) was 1.62 (95% CI, 1.43–1.84; *P* < .01), indicating that the risk of being tested for *C. difficile* continued to be elevated after the conclusion of antibiotic therapy. For patients on high-risk postdischarge antibiotics, the hazard ratio compared to patients not on postdischarge antibiotics was 1.56 (95% CI, 1.42–1.71; *P* < .01) and the hazard ratio for postantibiotic exposure among these patients was 2.39 (95% CI, 2.07–2.76; *P* < .01). Other factors associated with a significantly higher hazard for *C. difficile* testing included gastric acid suppression, the duration of inpatient antibiotic exposure, being on immunosuppressive medications, and having certain comorbidities (Table 2).

**Table 2.** Multivariable Cox regression model to estimate the outcome of *C. difficile* testing

Characteristic	Hazard ratio (95% CI)	*p* -Value
**Age**	1.00 (1.00–1.00)	<.01
**Male sex**	0.83 (0.77–0.89)	<.01
**Medicine service at discharge**	1.02 (0.98–1.07)	.31
**Length of stay (days)**	1.00 (1.00–1.00)	.81
**Inpatient antibiotic exposure**		
DOT for agents with the highest risk for *C. difficile*	1.03 (1.03–1.04)	<.01
DOT for all other antibiotics	1.03 (1.02–1.03)	<.01
**Postdischarge antibiotic exposure, relative to no antibiotic exposure**		
Low-risk antibiotic exposure	1.40 (1.29–1.51)	<.01
High-risk antibiotic exposure	1.56 (1.42–1.71)	<.01
Postantibiotic effect, low-risk antibiotics	1.62 (1.43–1.84)	<.01
Postantibiotic effect, high-risk antibiotics	2.39 (2.07–2.76)	<.01
**Gastric acid suppression**	1.25 (1.21–1.30)	<.01
**Body mass index, relative to normal** ^1^		
Underweight	1.13 (1.02–1.24)	.02
Overweight	0.95 (0.91–1.00)	.05
Obese	0.94 (0.90–0.99)	.01
Missing	0.78 (0.72–0.85)	<.01
**Immunosuppressive medication**	1.78 (1.38–2.30)	<.01
**Comorbidities**		
Alcohol use disorder	1.01 (0.96–1.06)	.64
Anemia	1.30 (1.25–1.36)	<.01
Cardiac arrhythmia	1.04 (1.00–1.08)	.04
Chronic obstructive pulmonary disease	1.00 (0.96–1.03)	.89
Chronic pancreatitis	1.47 (1.33–1.63)	<.01
Congestive heart failure	0.95 (0.91–0.99)	.02
Diabetes mellitus	1.11 (1.07–1.15)	<.01
Inflammatory bowel disease	2.78 (2.58–2.99)	<.01
Renal disease or dialysis	1.33 (1.28–1.39)	<.01
Immunosuppressive condition^2^	2.03 (1.92–2.14)	<.01
Liver disease	1.26 (1.20–1.31)	<.01
Metastatic cancer	1.59 (1.50–1.69)	<.01
Neurological disorders^3^	1.24 (1.19–1.29)	<.01
Pulmonary circulation disorders	1.09 (1.02–1.17)	.02
Peripheral vascular disease	1.22 (1.17–1.27)	<.01
Weight loss	1.52 (1.45–1.59)	<.01

Abbreviations: DOT days of therapy.

1. Body mass index was classified as underweight (<18.5), normal (18.5–24.9), overweight (25.0–29.9) obese (30 and higher), and missing.

2. Immunocompromising diagnoses included lymphoma, leukemia, HIV, and organ transplantation.

3. This category included dementia, paralysis, paresis, Parkinson’s disease, multiple sclerosis, epilepsy, and other neurological disorders.

The hazard ratio of being tested for *C. difficile* while on high-risk vs low-risk antibiotics was 1.12 (95% CI, 1.00–1.25, *P* = .06). The hazard ratio for being tested for *C. difficile* during the 7 days after the conclusion of high-risk vs low-risk antibiotics was 1.32 (95% CI, 1.15–1.51; *P* < .01).

In both subset analyses (patients ≥ 65 yr of age and use of gastric suppressants), the hazard ratio for *C. difficile* testing while on postdischarge antibiotics was comparable to the primary analysis. These results are shown in Supplemental Tables 1 and 2.

## Discussion

Antibiotics are commonly prescribed at hospital discharge and are often unnecessary or suboptimal.^2–6^ In this study, we have shown that patients who received postdischarge antibiotics were 40–56% more likely to be tested for *C. difficile* than those who did not receive antibiotics at discharge. This elevated risk persisted for at least 7 days after postdischarge antibiotics were completed. However, the overall frequency of testing across all patients was low, occurring in approximately 1 out of 100 patients during the 30 days after hospital discharge.

We suspect that the reason for increased *C. difficile* testing among those on postdischarge antibiotics was a greater incidence of clinically significant diarrhea, which likely was antibiotic-associated. However, we cannot rule out the possibility that providers may have simply had a heightened suspicion for *C. difficile* infection given the active or recent use of antibiotics in these patients; such a heightened suspicion may have lowered the providers’ threshold for ordering a *C. difficile* test in the context of loose stools. Either way, exposure to postdischarge antibiotics was associated with more diarrhea and/or a greater need for *C. difficile* testing during the 30 days after a hospital stay. These are both meaningful outcomes that can likely be prevented through more judicious antibiotic-prescribing at the point of hospital discharge.

In a study of patients hospitalized with pneumonia across 43 hospitals in Michigan, each excess day of postdischarge antibiotic treatment was associated with a 5% increase in the odds of an antibiotic-associated adverse event. The most common side effects were diarrhea, gastrointestinal distress, and mucosal candidiasis.^15^ Similarly, our study also found an increased risk of antibiotic-associated diarrhea for each day a patient was on postdischarge antibiotics, but we were unable to quantify how many of these antibiotics were indicated or unnecessary.

An ongoing challenge with antibiotic stewardship is demonstrating antibiotic-related harm. Harm, when it does occur, is often not observed by the provider who prescribed the antibiotics either because the harm goes unrecognized or because it occurs when the patient is no longer under that prescriber’s care.^16^ Antibiotic resistance, for example, may develop weeks to months after an antibiotic is prescribed.^17,18^ If antibiotic resistance does develop and is detected, this information may never reach the initial prescriber. Therefore, there is a need for strategies on leveraging real antibiotic-related harms to motivate providers to improve their use of antibiotic agents.^19^ We hope our findings are helpful in this regard.

Our study is not without its limitations. First, we were unable to capture *C. difficile* testing done outside VHA, so we may have undercounted the frequency at which this outcome occurs. However, because the likelihood of being tested outside the VHA probably did not differ based on post-discharge antibiotic use, this missing data is unlikely to have biased our results. Second, by using *C. difficile* testing to measure our outcome, we likely underestimated the incidence of diarrhea in our cohort, as clinicians who had a reasonable alternative diagnosis for the patient’s diarrhea may not have ordered a *C. difficile* test. Limiting our cohort to patients discharged to the community may have also reduced the frequency of the outcome. Third, patients may have been prescribed additional outpatient antibiotics during the 30-day window period, and these antibiotics may have contributed to their risk of being tested for *C. difficile*. Capturing these additional antibiotics, both from VHA and non-VHA sources, was beyond the scope of this study. Fourth, we only could measure which antibiotics were dispensed at discharge, not what the patient actually took or whether the prescribed antibiotic was appropriate in its selection, dosing or duration. Fifth, we were unable to capture outpatient intravenous antibiotics prescribed at discharge; based on our prior work, outpatient intravenous antibiotics were prescribed to only 2% of all patients discharged to the community, so our inability to capture this exposure likely had a minimal effect on our findings.^20^ Sixth, we only measured the hazard ratio during the 30 days after discharge because that period represents the time of greatest risk for *C. difficile* testing. However, the risk of developing healthcare-associated *C. difficile* likely extends beyond that 30-day window.^21^ Sixth, the postantibiotic effect was only measured during the 7 days after postdischarge antibiotics were stopped even though disturbances to the gut microbiome likely persisted for much longer.^22^ Finally, our findings may not be generalizable to non-VHA settings.

In conclusion, we found that the likelihood of *C. difficile* testing was 40% to 56% higher among patients prescribed postdischarge antibiotics compared to those without postdischarge antibiotic exposure. Efforts to optimize antibiotic-prescribing at hospital discharge, particularly by reducing excessive duration and avoiding high-risk agents, may help mitigate these risks.

## Supporting information

10.1017/ash.2025.10162.sm001 Livorsi et al. supplementary material Livorsi et al. supplementary material

## Supplementary material

The supplementary material for this article can be found at https://doi.org/10.1017/ash.2025.10162.
