# Supplementary material for: Association between postdischarge antibiotic use and C. difficile testing as a surrogate for clinically significant diarrhea
Source: Antimicrob Steward Healthc Epidemiol. 2025 Oct 16;5(1):e272. doi: 10.1017/ash.2025.10162 (PMC12538357; doi:10.1017/ash.2025.10162)
Supplement: Livorsi et al. supplementary material [file S2732494X25101629sup001.docx]

## **Supplemental Table 1. Multivariable Cox regression model to estimate the outcome of *C. difficile* testing among patients ≥ 65 years of age (n=1,117,262)**

| **Characteristic** | **Hazard Ratio (95% CI)** | **p-value** |
| --- | --- | --- |
| **Age** | 1.00 (1.00-1.00) | 0.75 |
| **Male sex** | 0.86 (0.76-0.97) | 0.01 |
| **Medicine service at discharge** | 1.02 (0.96-1.08) | 0.51 |
| **Length of stay (days)** | 1.00 (1.00-1.00) | 0.43 |
| **Inpatient antibiotic exposure**  DOT for agents with the highest risk for *C. difficile*  DOT for all other antibiotics | 1.04 (1.03-1.04)  1.03 (1.03-1.03) | <0.01  <0.01 |
| **Post-discharge antibiotic exposure, relative to no antibiotic exposure**  Low-risk antibiotic exposure  High-risk antibiotic exposure  Post-antibiotic effect, low-risk antibiotics  Post-antibiotic effect, high-risk antibiotics | 1.41 (1.28-1.54)  1.53 (1.36-1.71)  1.65 (1.42-1.92)  2.27 (1.91-2.70) | <0.01  <0.01  <0.01  <0.01 |
| **Gastric acid suppression** | 1.24 (1.19-1.29) | <0.01 |
| **Body Mass Index, relative to normal**^1^  Underweight  Overweight  Obese  Missing | 1.07 (0.95-1.20)  0.95 (0.90-1.01)  0.93 (0.88-0.99)  0.80 (0.73-0.89) | 0.27  0.09  0.02  <0.01 |
| **Immunosuppressive medication** | 1.97 (1.43-2.71) | <0.01 |
| **Comorbidities**  Alcohol use disorder  Anemia  Cardiac arrhythmia  Chronic pancreatitis  Chronic obstructive pulmonary disease  Congestive heart failure  Diabetes mellitus Immunosuppressive condition^2^  Inflammatory bowel disease  Liver disease  Metastatic cancer  Neurological disorders^3^  Pulmonary circulation disorders  Peripheral vascular disease  Renal disease or dialysis  Weight loss | 1.03 (0.97-1.09)  1.29 (1.23-1.35)  1.02 (0.98-1.07)  1.53 (1.33-1.77)  0.98 (0.94-1.02)  0.98 (0.93-1.02)  1.11 (1.06-1.16)  1.92 (1.80-2.05)  2.44 (2.22-2.69)  1.21 (1.15-1.28)  1.58 (1.48-1.69)  1.25 (1.19-1.31)  1.08 (1.00-1.18) 1.19 (1.14-1.25)  1.30 (1.25-1.36)  1.49 (1.42-1.58) | 0.38  <0.01  0.37  <0.01  0.33  0.32  <0.01  <0.01  <0.01  <0.01  <0.01  <0.01  0.05  <0.01  <0.01  <0.01 |

Abbreviations: DOT days of therapy

- 1. Body mass index was classified as underweight (<18.5), normal (18.5-24.9), overweight (25.0-29.9) obese (30 and higher), and missing.
  2. Immunocompromising diagnoses included lymphoma, leukemia, HIV, and organ transplantation.
  3. This category included dementia, paralysis, paresis, Parkinson’s disease, multiple sclerosis, epilepsy, and other neurological disorders.

## **Supplemental Table 2. Multivariable Cox regression model to estimate the outcome of *C. difficile* testing among patients receiving proton pump inhibitors and/or histamine H2-receptor antagonists during the hospital stay are at the time of hospital discharge (n=799,581)**

| **Characteristic** | **Hazard Ratio (95% CI)** | **p-value** |
| --- | --- | --- |
| **Age** | 1.00 (1.00-1.00) | 0.26 |
| **Male sex** | 0.84 (0.76-0.92) | <0.01 |
| **Medicine service at discharge** | 1.12 (1.05-1.19) | <0.01 |
| **Length of stay (days)** | 1.00 (1.00-1.00) | 0.37 |
| **Inpatient antibiotic exposure**  DOT for agents with the highest risk for *C. difficile*  DOT for all other antibiotics | 1.03 (1.02-1.04)  1.02 (1.02-1.03) | <0.01  <0.01 |
| **Post-discharge antibiotic exposure, relative to no antibiotic exposure**  Low-risk antibiotic exposure  High-risk antibiotic exposure  Post-antibiotic effect, low-risk antibiotics  Post-antibiotic effect, high-risk antibiotics | 1.34 (1.21-1.48)  1.47 (1.29-1.66)  1.65 (1.40-1.95)  2.23 (1.83-2.70) | <0.01  <0.01  <0.01  <0.01 |
| **Body Mass Index, relative to normal**^1^  Underweight  Overweight  Obese  Missing | 1.15 (1.01-1.31)  0.95 (0.89-1.01)  0.95 (0.89-1.02)  0.78 (0.69-0.87) | 0.03  0.09  0.14  <0.01 |
| **Immunosuppressive medication** | 2.31 (1.75-3.05) | <0.01 |
| **Comorbidities**  Alcohol use disorder  Anemia  Cardiac arrhythmia  Chronic pancreatitis  Chronic obstructive pulmonary disease  Congestive heart failure  Diabetes mellitus Immunosuppressive condition^2^  Inflammatory bowel disease  Liver disease  Metastatic cancer  Neurological disorders^3^  Pulmonary circulation disorders  Peripheral vascular disease  Renal disease or dialysis  Weight loss | 1.05 (0.99-1.12)  1.24 (1.18-1.31)  1.06 (1.01-1.11)  1.40 (1.25-1.58)  0.98 (0.94-1.03)  0.96 (0.91-1.02)  1.09 (1.04-1.15)  2.02 (1.88-2.17)  2.47 (2.24-2.71)  1.22 (1.15-1.29)  1.52 (1.41-1.64)  1.25 (1.19-1.32)  1.09 (1.00-1.19) 1.18 (1.12-1.25)  1.32 (1.26-1.39)  1.54 (1.45-1.66) | 0.13  <0.01  0.02  <0.01  0.43  0.19  <0.01 <0.01  <0.01  <0.01  <0.01  <0.01  0.05  <0.01  <0.01  <0.01 |

Abbreviations: DOT days of therapy

- 1. Body mass index was classified as underweight (<18.5), normal (18.5-24.9), overweight (25.0-29.9)

obese (30 and higher), and missing.

- 1. Immunocompromising diagnoses included lymphoma, leukemia, HIV, and organ transplantation.
  2. This category included dementia, paralysis, paresis, Parkinson’s disease, multiple sclerosis, epilepsy, and other neurological disorders.
